# A Practical Data Recovery Technique for Long-Term Strain Monitoring of Mega Columns during Construction

**DOI:** 10.3390/s130810931

**Published:** 2013-08-19

**Authors:** Se Woon Choi, EunMi Kwon, Yousok Kim, Kappyo Hong, Hyo Seon Park

**Affiliations:** Department of Architectural Engineering, Yonsei University, Seoul 110-732, Korea; E-Mails: watercloud@yonsei.ac.kr (S.W.C.); eunmi228@yonsei.ac.kr (E.K.); yskim1220@yonsei.ac.kr (Y.K.); kappyo@yonsei.ac.kr (K.H.)

**Keywords:** strain sensing, wireless sensor system, vibrating wire strain gages, structural health monitoring

## Abstract

A practical data recovery method is proposed for the strain data lost during the safety monitoring of mega columns. The analytical relations among the measured strains are derived to recover the data lost due to unexpected errors in long-term measurement during construction. The proposed technique is applied to recovery of axial strain data of a mega column in an irregular building structure during construction. The axial strain monitoring using the wireless strain sensing system was carried out for one year and five months between 23 July 2010 and 22 February 2012. During the long-term strain sensing, three different types of measurement errors occurred. Using the recovery technique, the strain data that could not be measured at different intervals in the measurement were successfully recovered. It is confirmed that the problems that may occur during long-term wireless strain sensing of mega columns during construction could be resolved through the proposed recovery method.

## 1. Introduction

As the shapes of building structures become irregular or their height and size increase, mega columns, whose cross sectional area is larger than that of conventional members, are used more frequently. A mega column, a vertical member that comprises the resisting system against a lateral load such as earthquake or wind load, is placed at perimeter of a building plane in order to efficiently control the lateral displacement of the structure [1,2].

For structures in which relatively large mega columns have been used, the safety of the members are managed during construction through structural health monitoring (SHM) using various sensors, including axial strain sensors [3–5]. Various sensing methods have been developed for field measurements of strains in buildings and infrastructures [6–10]. Recent significant developments of new strain sensors and their applications can be found in [11–14]. A wireless sensor network is preferred for the SHM of large-scale structures under construction because of the potential instability of the electricity supply and possibility of unexpected damage to the sensors and transceiver lines, and the real-time evaluation of the safety of structural members should be taken into account [15,16].

However, it is difficult for a wireless sensor network system not to lose data measured by the sensors for various unexpected reasons such as communication problems, noise, failure or abnormal installation of the sensors, harsh weather conditions, and hardware failures [17,18]. Various studies on the recovery of lost data have mostly been focused on the failure tolerance of the wireless sensor networks [19,20]. The concept of large-scale neuron sensor networks or a carbon nanotubes (CNT)-based artificial neural system (ANS) [11,12,21] or the use of artificial neural networks in management of strain data in a sensor network [22,23] may be used in estimation of strain data without recovery of abnormal sensors.

In this study, a practical data recovery method is proposed for the data loss that occurred during the safety monitoring of mega columns in a large-scale irregular structure that was actually under construction. The on-site safety monitoring of the constructed mega columns was accomplished by measuring the longitudinal strains with four wireless vibrating wire strain gauges (VWSG) installed vertically on the same cross section [15]. In this study, the analytical correlation among the longitudinal strains measured by the four wireless VWSG sensors was derived for the recovery of lost data. Based on the correlations, the strain data that could not be measured at each of the measurement intervals in the measurement data for a total of one year and five months between 23 July 2010 and 2 February 2012 were recovered, and the errors contained in the data measured by the damaged sensors were modified.

## 2. Strain Sensing System for a Mega Column in the Irregular Building under Construction

**Figure 1.**
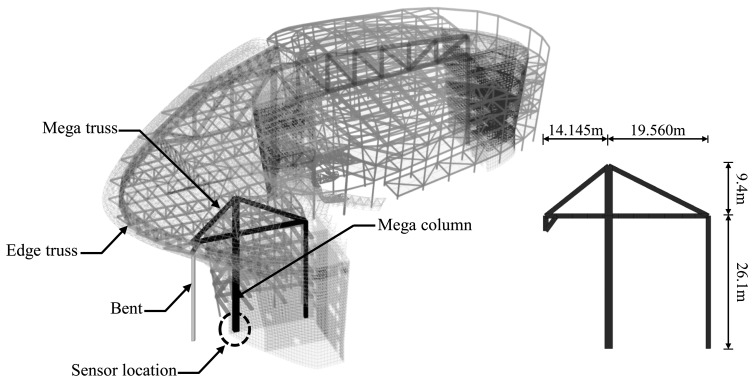
Mega column in a large-scale irregular building structure.

A practical monitoring system is applied to wireless strain sensing of the mega column in an irregular building (Figure 1) composed of three basement levels and four levels above the ground. As shown in Figure 1, a 26.1 m long mega column is used to support the mega truss. Since the structure is mainly used for exhibitions, large-scale structural members such as mega columns and mega trusses were employed to secure sufficiently large open spaces.

Due to the advantages of simplicity in the operating principles and the low cost of installation compared to other sensors, VWSGs are the most frequently used sensors in the construction field and for static strain monitoring of building structures. In this study, Geokon's VSM-400 model [24] with a length of 150 mm as the VWSG was used, installed at the centers of the section of the mega column on the fourth basement level of the irregular building shown in Figure 1.

**Figure 2.**
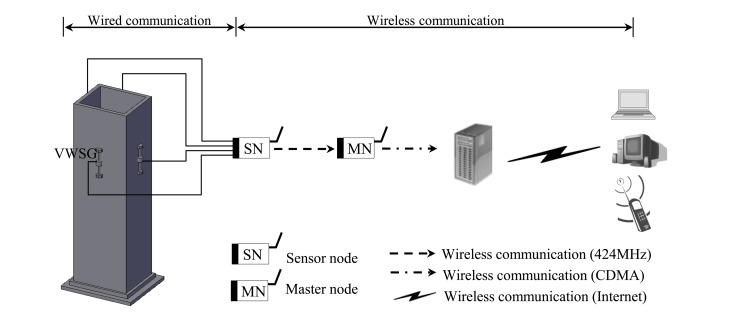
Wireless strain sensing system for the mega-column based on VWSG.

The measurement system for the sensing of longitudinal strain of the mega column consisted of four VWSGs installed on the mega column, a sensor node that transmits the information measured by the sensors to the master node through a wireless transmission system, and a master node that transmits all the measurement data received from the sensor node to the monitoring server through the long distance wireless transmission system shown in Figure 2.

**Figure 3.**
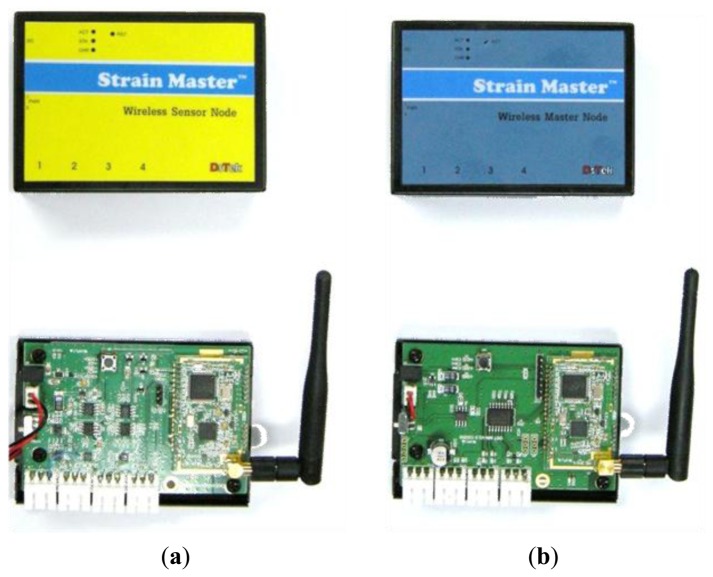
Sensor node and master node for wireless sensing system. (**a**) Sensor node (**b**) Master node.

As shown in Figure 3a, the wireless sensor node for the 4-channel VWSG system consists of a sensor processing module, processor, memory, communication module, and power supply unit [15]. During construction, the sensor node inevitably requires batteries where continuous power supply through a wire is impossible. The low power consumption technology (LPCT) was adopted to resolve the power supply problem. The LPCT is realized by controlling the nodes through the active-sleep mode. In active mode, a sensor node performs every function. To minimize power consumption, sleep mode makes a sensor node inactive, which means that transmitting and receiving of the data is disabled. In addition, the transceiver and peripheral circuit are inserted into a single chip (CC1020 transceiver by Chipcon) which is used in communication with 424 MHz UHF within the Industrial Scientific and Medical (ISM) bandwidth, to operate with low power consumption [25]. In this study, the sensor node is designed to consume an electric current of 50 mA in active mode and 200 μA in sleep mode. In addition, the sensor nodes are also operated by external DC power.

A master node (Figure 3b) serves the role of transmitting the data to the monitoring server located at a long distance by mobile communication after receiving the measured values from sensor nodes. A master node also has power issues. Thus, LPCT with active-sleep mode, which is already applied in the sensor nodes, was also adopted in the master node. If sensor nodes are in active mode, but the master node is in sleep mode, data cannot be transmitted to a monitoring server. Thus, the setting of active-sleep mode among sensor-master node should be in identical manner for successful transmission.

The data is transmitted between the master node and the monitoring server by some mobile communication technology. The master node consists of two parts; a receiver module and Code-Division Multiple Access (CDMA) transmitter. The receiver module of the master node is directly connected with a commercially available CDMA transmitter through a short cable. After the receiver module receives and delivers the measurement data from the sensor nodes to the cable, the data is transmitted at the CDMA transmitter by means of the mobile communication system, as shown in Figure 2.

## 3. Analytical Relationship among Measured Longitudinal Strains

**Figure 4.**
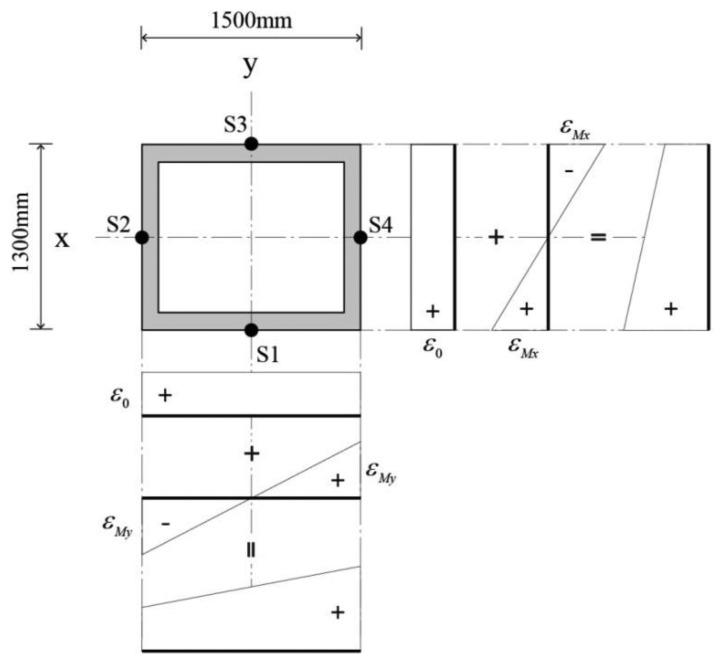
Stress distribution of the mega column subjected simultaneously to an axial force and bending moments.

The longitudinal strain *ε_i_* (*i* = 1 to 4) measured at the four sensors of S1, S2, S3, and S4 installed on the cross section of a mega column in Figure 4 can be expressed as the sum of the strains generated by the axial force, *ε_0_*, and the strains generated by the bending moment applied on the x and y axes, *ε_Mx_* and *ε_My_*. Since the strain due to the bending moment is proportional to the distance from the neutral axis, a compression strain and a tensile strain of the same magnitude are generated simultaneously. In Figure 4, the compression strain was denoted as positive while the tensile strain is negative. In the case where individual sensors are installed at the neutral axis of the cross section, the effect of the biaxial bending moment does not need to be taken into consideration; thus, the effect of the bending moment with respect to the y axis ±*ε_My_* is negligible for the sensors of S1 and S3. Likewise, only the effect of the bending moment with respect to the y axis needs to be taken into consideration for the strain measured at the sensors S2 and S4. Table 1 shows the combined stresses at each sensor location.

In such cases, three unknown variables are needed to determine the strain at the arbitrary sensors installed on the mega column: *ε*_0_, *ε_Mx_*, and *ε_My_*. Hence, the three unknowns may be determined by randomly choosing three out of the four sensors. With the measurements from the sensors of S1 and S3 or those from the sensors of S2 and S4, the strain due to the axial force, *ε*_0_, can be obtained from Equation (1):

(1) $$\varepsilon_{0}=\frac{\varepsilon_{1}+\varepsilon_{3}}{2}=\frac{\varepsilon_{2}+\varepsilon_{4}}{2}$$

If *ε*_0_ is calculated using the strains *ε*_1_ and *ε*_3_ in Equation (1), then *ε_Mx_* can be expressed as in Equation (2), and *ε_My_* as in Equation (3) or Equation (4):

(2) $$\varepsilon_{Mx}=\varepsilon_{1}-\varepsilon_{0}=\varepsilon_{0}-\varepsilon_{3}=\frac{\varepsilon_{1}-\varepsilon_{3}}{2}$$

(3) $$\varepsilon_{My}=\varepsilon_{0}-\varepsilon_{2}=\frac{\varepsilon_{1}+\varepsilon_{3}}{2}-\varepsilon_{2}$$

(4) $$\varepsilon_{My}=\varepsilon_{4}-\varepsilon_{0}=\varepsilon_{4}-\left(\frac{\varepsilon_{1}+\varepsilon_{3}}{2}\right)$$

In the same manner, if *ε*_0_ is calculated using the strains *ε*_2_ and *ε*_4_ in Equation (1), then *ε_Mx_* can be expressed as in Equation (5) or Equation (6), and *ε_My_* as in Equation (7):

(5) $$\varepsilon_{Mx}=\varepsilon_{1}-\varepsilon_{0}=\varepsilon_{1}-\frac{\varepsilon_{2}-\varepsilon_{4}}{2}$$

(6) $$\varepsilon_{Mx}=\varepsilon_{0}-\varepsilon_{3}=\frac{\varepsilon_{2}+\varepsilon_{4}}{2}-\varepsilon_{3}$$

(7) $$\varepsilon_{My}=\varepsilon_{0}-\varepsilon_{2}=\varepsilon_{4}-\varepsilon_{0}=\frac{\varepsilon_{4}-\varepsilon_{2}}{2}$$

When Equations (1)–(7) are used, even if the measurements have not been normally carried out by one of the four sensors installed to the four sides of the mega column, the non-measured data is recovered based on the strain data normally measured by the other three sensors. For instance, if the strain *ε*_1_ was not measured at the sensor S1, the axial strain *ε*_0_ can be calculated as the mean of the strains *ε*_2_ and *ε*_4_ measured at the sensors S2 and S4. With the strains from the sensors of S2 and S4, the remaining unknowns *ε_Mx_* and *ε_My_* can be obtained from Equations (6) and (7), respectively. Then, the lost measurement data, *ε*_1_, can be recovered according to the first row of Table 1.

## 4. Application to Recovery of Longitudinal Strains in Mega Columns

### 4.1. Measurement of Axial Strain during Construction

**Figure 5.**
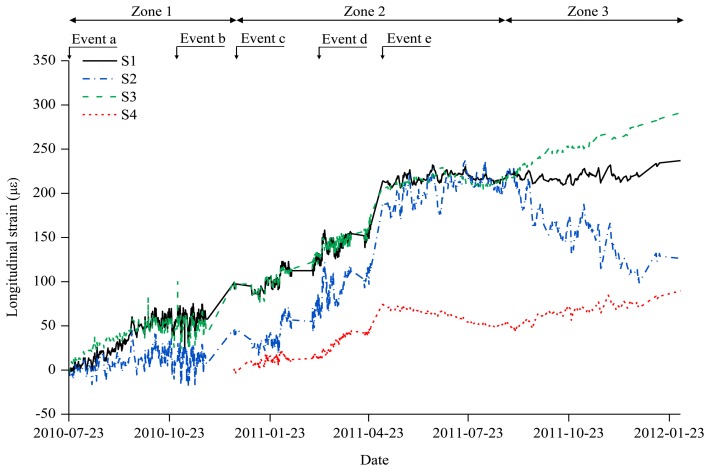
Measurement histories of axial strains in the mega-column during construction.

During the construction of the irregular building structure in Figure 1, axial strain sensing using the wireless VWSG-based strain sensing system in Figure 2 was carried out for one year and five months between 23 July 2010, and 22 February 2012. The plot in Figure 5 shows the measurement values over the total sensing period of the four VWSGs, S1–S4, installed on the mega column. A total of 21,808 measurement values were obtained from the four VWSGs during the sensing period. For the monitoring, the distinctive procedures that took place during the sensing period can be classified into five types of events:

a. Installation of VWSGs (S1,S2,S3)
b. Installation of the edge truss block, 30 October 2010
c. Installation of VWSG S4, 20 December 2010
d. Pouring concrete on the 4^th^ floor cantilever slab, 11 March 2011
e. Removal of supporters (bend in Figure 1) for the edge truss, 25 April 2011

As shown in Figure 5, the structural response of the mega-column is directly observed through the changes in the measurement values caused by the procedures during the construction period, including the addition of materials and increase of the self-loading, which were reflected by the measurement values and plots obtained from monitoring.

The overall monitoring period was divided into three zones with reference to the major events. Zone 1 is the interval in which, among the four measurement sensors, the measurement sensor S4 was not installed from 23 July 2010 to 20 December 2010. Zone 2 is the interval from 20 December 2010 to 28 August 2011 during which all the measurement sensors (S1∼S4) operated normally. Finally, Zone 3 is the interval in which there was an anomaly in the S2 measurement sensor from 28 August 2011 to 22 February 2012.

### 4.2. Recovery of Strain Data

In this study, the measurement data of the S4 sensor, which was not installed in Zone 1, were reconstructed with the data of the other three installed sensors. In Zone 2, the S4 sensor measurement data, which were newly started for measurements, were converted into the strains accumulated from the initial measurements using the S4 data reconstructed in Zone 1. The normally obtained data of the four VWSGs were used to compare the values of one sensor predicted on the basis of the data of three randomly chosen sensors with their actual measurement values. In Zone 3 in which the S2 sensor showed an anomaly, the S2 sensor values were modified using the data of the S1, S3, and S4 sensors.

#### 4.2.1. Zone 1

**Figure 6.**
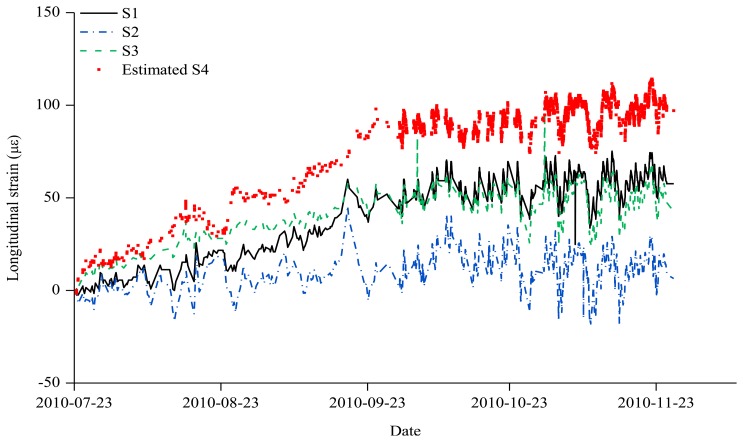
Estimated data for S4 and measurement data for S1, S2, and S3 (Zone 1).

Only three sensors out of a total of four sensors were installed in Zone 1 during the entire sensing period. Hence, the strain *ε*_4_ at the S4 sensor location, which could not be measured, was estimated. To determine the strain values of the S4 sensor that was not installed, the values of *ε*_0_ and *ε_My_* were required, as shown in the fourth row of Table 1. As in Equation (1), *ε*_0_ is calculated as the mean of *ε*_1_ and *ε*_3_. According to Equation (3), *ε_My_* is determined with the values of *ε*_1_, *ε*_2_, and *ε*_3_. The strain values at the S1, S2, and S3 sensors as well as the *ε*_0_, *ε_My_*, and *ε*_4_ values estimated using the strain values at the three sensors are shown in Table 2 at monthly intervals from the first day of the sensing. Figure 6 shows the S1–S3 measurements that were obtained through the constant monitoring and the S4 estimations during the period from 23 July 2010, the day when the monitoring started, to the time when the S4 sensor was installed.

#### 4.2.2. Zone 2

**Figure 7.**
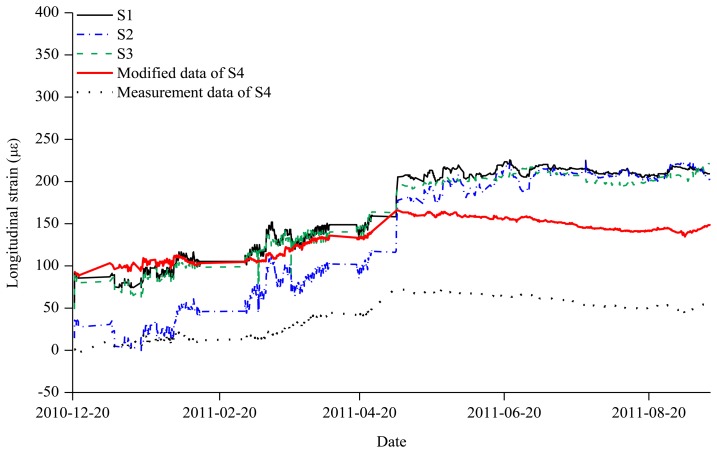
Measured strains from S1, S2, S3, and S4 and modified strain by adding the accumulated strain of 91.52 *με* for S4 (Zone 2).

Figure 7 shows the measurements of the S4 sensor that was installed on 20 December 2010, about five months after the start of the measurements, and the measurements of the sensors S1–S3 in Zone 2. The period represented by Zone 2 is from 20 December 2010, when sensor S4 was installed, to 28 August 2011, when sensor S2 showed an abnormality. The measurements of S4 were different from those of the S1–S3 sensors that had initially been installed. It is necessary to modify the measurement data of S4 in Zone 2 into the strain accumulated from the initial measurement.

The starting value of S4 in Zone 2, estimated through the S1, S2, and S3 measurements in Zone 2, was compared with the final value of S4 in Zone 1 estimated using the S1, S2, and S3 measurements in Zone 1. The estimated value for S4 value in Zone 1 was 91.52 *με*, and that in Zone 2 was 96.31 *με*. With the consistency of about 95% in the two values, the measurement data on the day of 20 December 2010 when the S4 sensor was installed were transferred in parallel as much as the S4 value which was 91.52 *με* In Figure 7, the S4 measurement values in Zone 2 were modified by considering the estimation of the S4 sensor at the last period of Zone 1.

The variance accounted for (VAF) in Equation (8) was applied to investigate the accuracy of the estimations and measurements [26,27]:

(8) $$\text{VAF}=\left[1-\frac{\mathrm{var}(y_{me}-y_{es})}{\mathrm{var}(y_{me})}\right]\times 100$$

The estimated and measured data are denoted as *y_es_* and *y_me_*, respectively. “var” denotes variance. Table 3 shows the result of the quantitative evaluation of the estimations and measurements, indicating that the values of S1, S2, and S3 are all consistent by more than 98%.

**Figure 8.**
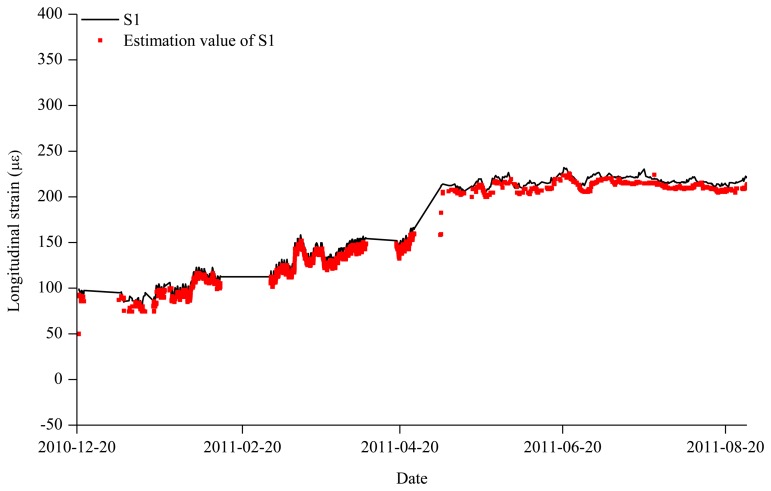
Comparison of estimated and measured strain data for S1 (Zone 2).

In Figure 8, for example, the estimated strain data based on the S2, S3, and S4 sensors are compared the measured data from S1. It is found that the estimated data matched well with the measured data.

#### 4.2.3. Zone 3

**Figure 9.**
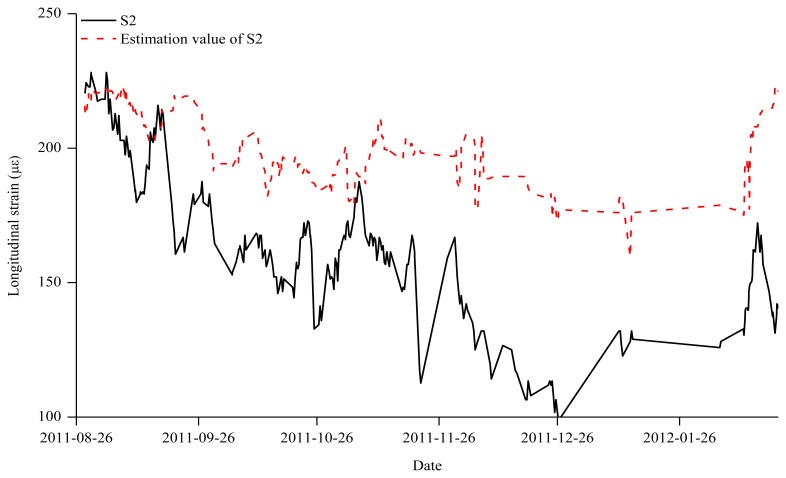
Estimated and measured strain data for S2 (Zone 3).

**Figure 10.**
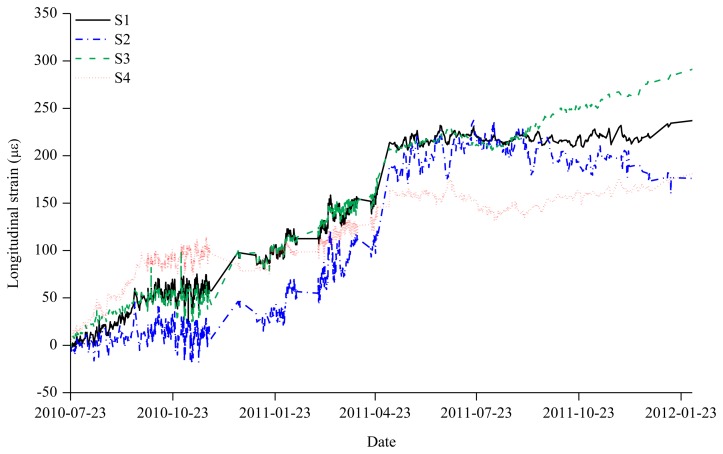
Strain histories for the mega column based on the recovery technique during construction.

In Zone 3, where the anomaly of the S2 sensor was found, increases in the measurements due to the increased load were found in the S1, S3, and S4 sensors. However, the measurements of the S2 sensor were decreasing abnormally in Zone 3 as shown in Figure 9. The reason for the S2 measurement anomaly is not known. Thus, in Figure 9, the S2 value was estimated on the basis of the measurement data of the S1, S3 and S4 sensors that were normally operating so that the estimated S2 value could replace the previous measurement data. Finally, Figure 10 shows the estimation values of the S4 sensor that was not installed in Zone 1, the parallel transfer of the S4 value in Zone 2, and the modified measurement of the abnormal S2 sensor in Zone 3.

## 5. Conclusions

In this study, a strain data recovery technique is proposed for the data losses during structural health monitoring of a mega column in a large-scale irregular building structure. The monitoring was done for one year and five months. During the monitoring, problems such as non-installation of a measurement sensor and abnormal sensor operation occurred. The entire period was divided into three zones: Zone 1 when the S4 sensor had not been installed, Zone 2 when the monitoring was performed normally because the S1–S4 sensors were installed and Zone 3 when the anomaly of the S2 sensor was found. Based on the analytical relations among the measured stains, the measurements of the S4 sensor, which had not been installed in Zone 1, were estimated with a simple estimation method. Additionally, the values measured after the installation of the S4 sensor were transferred in parallel as much as the S4 value measured in Zone 2, and then the structural response of the S1–S4 sensors was monitored. Finally, the measurement of the S2 sensor in which the anomaly was found in Zone 3 was replaced by the estimation based on the S1, S3 and S4 sensor measurements. The problems that may occur during long-term wireless sensor monitoring during construction could be resolved through the estimation method. In addition, the safety of structural members during the monitoring may be evaluated by estimating the axial force and moments of the structural members. It is noted that analytical correlation among the strains from strain sensors is required for the application of the data recovery method.

## Figures and Tables

**Table 1.** Combined strain at each sensor location.

**Sensor Location**	**Axial Strain**	**Bending Strain**	**Combined Strain**
S1	*ε*_0_	*ε_Mx_*	*ε*_1_=*ε*_0_+*ε_Mx_*
S2	*ε*_0_	−*ε_My_*	*ε*_2_=*ε*_0_ − *ε_My_*
S3	*ε*_0_	−*ε_Mx_*	*ε*_3_=*ε*_0_ − *ε_Mx_*
S4	*ε*_0_	*ε_My_*	*ε*_4_=*ε*_0_ + *ε_My_*

**Table 2.** Measured strains and estimated strain of *ε*_4_ in Zone 1.

**Time**	**Measured Strains** (*με*)			**Estimated Strains** (*με*)		**Combined Strain** (*με*)
		
	***ε*_1_**	***ε*_2_**	***ε*_3_**	*ε*_0_	***ε****_My_*	*ε*_4_
2010-07-23	0.00	0.00	0.00	0.00	0.00	0.00
2010-07-23	−1.62	−2.36	−1.58	−1.60	0.75	−0.85
2010-08-23	20.84	17.34	27.34	24.09	6.74	30.83
2010-08-23	20.07	14.99	28.11	24.09	9.10	33.19
2010-09-23	36.88	−5.48	40.13	38.51	43.99	82.50
2010-09-23	42.45	0.81	44.15	43.30	42.49	85.79
2010-10-24	69.58	34.61	57.72	63.65	29.04	92.69
2010-10-24	69.58	35.38	57.72	63.65	28.27	91.92
2010-11-26	57.64	6.34	42.53	50.08	43.75	93.83
2010-11-26	57.64	7.88	41.76	49.70	41.82	91.52

**Table 3.** The VAF of each sensor.

	**S1 (%)**	**S2 (%)**	**S3 (%)**
VAF of Zone 2	99.36	98.69	99.09
